# Correction to: COVID-19-related acute kidney injury; incidence, risk factors and outcomes in a large UK cohort

**DOI:** 10.1186/s12882-021-02617-2

**Published:** 2021-12-06

**Authors:** Paul D. Jewell, Kate Bramham, James Galloway, Frank Post, Sam Norton, James Teo, Richard Fisher, Rohit Saha, Sam Hutchings, Phil Hopkins, Priscilla Smith, Jennifer Joslin, Satish Jayawardene, Sarah Mackie, Ali Mudhaffer, Amelia Holloway, Henry Kibble, Mosammat Akter, Benjamin Zuckerman, Kieran Palmer, Ciara Murphy, Domniki Iatropoulou, Claire C. Sharpe, Eirini Lioudaki

**Affiliations:** 1 Renal Unit, King’s College Hospital NHS Foundation Trust, Denmark Hill, London, SE5 9RS UK; 2 Faculty of Life Sciences and Medicine, King’s College London, London, UK; 3 Centre for Rheumatic Disease, King’s College London, London, UK; 4 Department of Sexual Health and HIV, King’s College Hospital NHS Foundation Trust, London, UK; 5 Department of Neurosciences, King’s College Hospital NHS Foundation Trust, London, UK; 6 Department of Critical Care, King’s College Hospital NHS Foundation Trust, London, UK

**Correction to: BMC Nephrol 22, 359 (2021)**

**https://doi.org/10.1186/s12882-021-02557-x**

Following publication of the original article [1], we have been informed that the incorrect figures have been introduces during the typesetting process.

The original article has been corrected.

The publisher apologizes for any confusion caused.
